# Five Neuropeptide Ligands Meet One Receptor: How Does This Tally? A Structure-Activity Relationship Study Using Adipokinetic Bioassays With the Sphingid Moth, *Hippotion eson*

**DOI:** 10.3389/fendo.2019.00231

**Published:** 2019-04-12

**Authors:** Heather G. Marco, Gerd Gäde

**Affiliations:** Department of Biological Sciences, University of Cape Town, Rondebosch, South Africa

**Keywords:** adipokinetic hormone, neuropeptide hormone, AKH receptor, *Hippotion eson*, lipid metabolism, structure-function assay, energy mobilization

## Abstract

Adipokinetic hormones (AKHs) play a major role in mobilizing stored energy metabolites during energetic demand in insects. We showed previously (i) the sphingid moth *Hippotion eson* synthesizes the highest number of AKHs ever recorded, viz. five, in its corpus cardiacum: two octa- (Hipes-AKH-I and II), two nona- (Hipes-AKH-III and Manse-AKH), and one decapeptide (Manse-AKH-II), which are all active in lipid mobilization (1). (ii) Lacol-AKH from a noctuid moth showed maximal AKH activity in *H. eson* despite sequence differences and analogs based on Lacol-AKH with modifications at positions 2, 3, 8, or at the termini, as well as C-terminally shortened analogs had reduced or no activity (2). Here we report on N-terminally shortened and modified analogs of the lead peptide, as well as single amino acid substitutions at positions 1, 4, 5, 6, and 7 by an alanine residue. Ala^1^ and Glu^1^ instead of pGlu are not tolerated well to bind to the *H. eson* AKH receptor, whereas Gln^1^ has high activity, suggesting it is endogenously cyclized. Replacing residue 5 or 7 with Ala did not alter activity much, in contrast with changes at position 4 or 6. Similarly, eliminating pGlu^1^, Leu^2^, or Thr^3^ from Lacol-AKH severely interfered with biological activity. This indicates that there is no core peptide sequence that can elicit the adipokinetic effect and that the overall conformation of the active peptide is required for a physiological response. AKHs achieve a biological action through binding to a receptor located on fat body cells. To date, one AKH receptor has been identified in any given insect species; we infer the same for *H. eson*. We aligned lepidopteran AKH receptor sequences and note that these are very similar. The results of our study is, therefore, also applicable to ligand-receptor interaction of other lepidopteran species. This information is important for the consideration of peptide mimetics to combat lepidopteran pest insects.

## Introduction

Neuropeptide hormones are biologically active peptides that are synthesized in modified neurons and released into the circulatory system to effect an action via a ligand-specific receptor. In insects, neurons of the corpus cardiacum (CC) synthesize and release neuropeptides that belong to the adipokinetic hormone (AKH)/red pigment-concentrating hormone (RPCH) family, so named for their classic action of mobilizing fuel metabolites from storage in the fat body of insects for catabolic purposes, and for their action on integumental pigment cells in crustaceans for camouflage (3). In insects the AKHs may have different chain lengths (octa-, nona-, and/or decapeptides), whereas the RPCHs in crustaceans only occur as octapeptides, to date, and in both animal groups, the ligand operates via a G protein-coupled receptor on the effector cell (4).

The AKH family of peptides has over 60 members, and some of these peptides can be common to different insect orders, for example Peram-CAH-I (one of the so-called cardioacceleratory hormones in *Periplaneta americana*) is synthesized in primitive insects (5), in cockroaches (6, 7), and in beetles (8, 9). On the other hand, certain AKHs are unique to one insect order, as is the case with currently nine order-specific AKH members (see Table 1) of the Lepidoptera, which comprises moths and butterflies. Many lepidopteran species (particularly in the larval stage of the insect lifecycle) are regarded as pest insects that compete for human food resources. One can envisage to produce a “green pesticide” that is harmful to lepidopterans but not to other insects, especially not to beneficial pollinator species, such as certain hymenopteran or dipteran (hoverfly) insects. The rationale of “green pesticides” in food security is to use information about endogenous hormones of pest insects to make peptide mimetics that will act only against the pest insects to alter their behavior or physiology (15). Thus, for such research it is paramount to know how the lepidopteran AKH receptor is activated by its ligands. In the current study we investigate such phenomena for the AKH receptor of a moth species. Here, we review data published previously (1, 2) and complement them with further analog studies emanating from 11 analogs not tested previously. The moth species of choice is the common striped hawk moth, *Hippotion eson*.

**Table 1:** Lepidoptera-specific Adipokinetic Hormone (AKH) family peptides.

**Name of peptide**	**Peptide sequence**	**Species (first elucidated)**	**References**
Hipes-AKH-I#	pELTFTSSWamide	*H. eson; Hippotion celerio*	(1)
Manse-AKH#	pELTFTSSWGamide	*Manduca sexta*	(10)
Lacol-AKH	pELTFTSSWGGamide	*Lacanobia oleracea*	(11)
Hipes-AKH-II#	pELTFTSTWamide	*H. eson; H. celerio*	(1)
Hipes-AKH-III#	pELTFTSTWGamide	*H. eson; H. celerio*	(1)
Bommo-AKH	pELTFTPGWGQamide	*Bombyx mori*	(11)
Manse-AKH-II#	pELTFSSGWGQamide	*M. sexta*	(12)
Helze-HrTH	pELTFSSGWGNamide	*Heliothis zea*	(13)
Piebr-AKH	pELTFSSGWamide	*Pieris brassicae*	(14)

# *Endogenous AKHs in Hippotion eson*.

Five years ago we demonstrated via electrospray mass spectrometry that *H*. *eson*, and the silver-striped hawk moth, *H. celerio*, each produce a record number of five different AKHs in their respective corpora cardiaca (1). The five AKHs are identical in these hawk moth species, and biological activity of the neuropeptides was examined with chemically synthesized peptides only in the more-abundant species, *H. eson*. The synthetic peptides were also used to confirm the sequence of the mature hawk moth AKHs [see Table 1; (1)]. All five peptides were active at a low dose to increase the circulating lipid concentration in resting *H. eson* adults, whereas the carbohydrate concentration was not significantly affected. Although not proven yet, it is highly likely that there is only one receptor for all five AKHs in *H. eson*. This has been the case in all other insects where a G protein-coupled receptor has been identified as the AKH receptor (AKHR), regardless of the number of AKH sequences encoded—see, for example, Marchal et al. (16) where AKHR information on *Aedes aegypti* with 1 ligand and *Schistocerca gregaria* with 3 ligands are shown. The AKHR of *H. eson*, is, therefore an interesting candidate to study with respect to ligand interactions, and could provide us with insight into what the structural limits are for adequate receptor-ligand interactions to effect a biological response. With this in mind, a functional study with *H. eson* was initiated when a decapeptide AKH from a water bug, Lacsp-AKH [pGlu-Val-Asn-Phe-Ser-Pro-Ser-Trp-Gly-Gly amide, (17)], with five amino acid substitutions compared with the endogenous decapeptide of the hawk moth, Manse-AKH-II, showed no adipokinetic activity in *H. eson* (1), whereas a decapeptide from another lepidopteran insect (Lacol-AKH: three amino acid substitutions compared with Manse-AKH-II; Table 1) was as active as the endogenous *H. eson* AKHs (2). The present study is a follow-on to elucidate the ligand criteria for the most effective functional response, i.e., an adipokinetic action. There is a tendency to evaluate the activity of neuropeptides only on the basis of their primary sequence and the charge of specific amino acids, whereas it is known that neuropeptides undergo conformational changes to obtain an “active conformation” once docking onto the receptor (18). In the present study we attempt to take such conformational change into account with our analog designs to determine the final activity and functional properties of a modified AKH in the sphingid moth. The AKH analogs are based on the sequence of Lacol-AKH (a Gly-extended version of the endogenous Manse-AKH of *H. eson*, see Table 1), and examine changes to the N-terminus of the peptide, including N-terminally shortened analogs, as well as a series of analogs with the single substitution by Ala in all positions of the peptide, excepting positions 2 and 3—substitutions in these positions were previously shown to have a profound negative effect on lipid-mobilization in *H. eson*, as did C-terminally shortened analogs (2).

## Materials and Methods

### Insects

Eggs and larvae of the common striped hawk moth, *H. eson*, on the leaves of the arum lily, *Zantedeschia aethiopica*, were collected during the austral months of May and June in 2015 and 2016 at the Buitenverwachting vineyard (Constantia, South Africa) and on the property of the University of Cape Town and reared at the University of Cape Town (Rondebosch, S. Africa). The material was placed in a constant temperature room of the Department of Biological Sciences and the insects were reared under the following conditions: 30°C, 60% RH, and a photocycle regime of 17 h light: 7 h dark. The larvae were reared on freshly cut arum lily leaves (in a jar of water) in a wire mesh cage with a wooden floor and dimensions of 45 × 40 × 45 cm (L × H × W). Daily, leaves were replaced and fecal matter was removed from the cage. Pupae were placed into individual small containers in the same rearing room. Under the described controlled holding conditions, adult moths eclosed between 11 and 12 days after pupation (H. G. Marco and G. Gäde, unpublished result) and were used in biological assays on the following day at room temperature.

This study was carried out in accordance with relevant institutional and national guidelines and regulations concerning the use of animal subjects in scientific studies.

### Biological Assay

A conspecific bioassay was carried out as described previously (1). Briefly, adult *H. eson* specimens of both sexes that had emerged during the night were used for biological assays on the following day. The cage of emerged moths were taken to the laboratory and left at RT for 1 h before sampling. During this time the moths were not active. Individual moths were removed from the cage one at a time: 0.5 μl hemolymph was sampled from the abdominal dorsal vessel and put into concentrated sulphuric acid; the moth was then injected ventro-laterally into the abdomen with 3 μl of either water, or the synthetic peptides under investigation (reconstituted in water to a concentration of 10 pmol in 3 μl), and the moth was put under a funnel at RT and left there until a second sample of hemolymph was taken 90 min after injection. The hemolymph was thoroughly mixed with the sulphuric acid, and the total vanillin-positive material (= lipids) was measured in the mixture as described previously (19). The number of moths per injectate ranged between 5 and 8 (see details of *n* number in Tables 2–**4**).

**Table 2:**
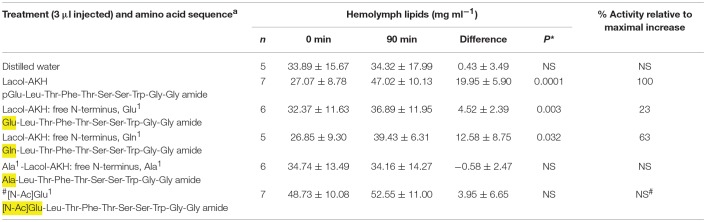
The biological effect of the N-terminus of Lacol-AKH in the common striped hawk moth, *Hippotion eson*.

*^a^Amino acid substitutions in Lacol-AKH analogs are highlighted*.

The data are presented as Mean ± S.D. ^#^Data from Marco and Gäde (2). ^*^Paired t-test was used to calculate the significance between pre-and post-injection values. NS, not significant.

The difference in lipid concentration before and after injection was calculated for each individual animal and a Student's *T*-test was used for calculating statistical significance in Excel.

### Synthetic Peptides

Peptides were custom-synthesized by Pepmic Co. Ltd (Suzhou, China). For primary structures see Tables 2–4. The synthetic peptides are based on a decapeptide code-named Lacol-AKH that was previously found as endogenous AKH in *Lacanobia oleracea* and *Mamestra brassicae* (11). Lacol-AKH is 100% active in *H. eson* as shown by *in vivo* lipid-mobilizing assays and was previously used in structure-activity studies with *H. eson* (2). Lacol-AKH differs in three places from the endogenous decapeptide in *H. eson*, i.e., Manse-AKH-II, which is also present in all sphingid moths investigated to date, and the first eight and nine amino acid residues of Lacol-AKH are, respectively, 100% identical to the endogenous octapeptide (Hipes-AKH-I) and nonapeptide (Manse-AKH) in *H. eson* [Table 1; (14)].

The rationale for the design of the synthetic peptides used in this study is the following: previously (2) we examined the effect of specific amino acid substitutions that cumulatively occur in Lacsp-AKH, a water bug AKH that is not active in *H. eson* (1), and found that the second (Leu) and third (Thr) amino acids are relatively important for biological activity in the sphingid moth, as well as Trp^8^. Here we have designed Ala-substituted analogs of Lacol-AKH to systematically examine the relative importance of charge and side-chains in positions 1 and 4–7. Additional analogs are designed to test the effect of Lacol-AKH without pGlu in position 1, and nonapeptides that differ N-terminally. Previously we looked at the effect of different peptide chain lengths on hyperlipemic activity *in vivo* by using analogs that were C-terminally extended, or truncated, or with a free C-terminus (2). In total, the current study investigates 11 different analogs for the first time (see Tables 2–4 for sequences).

### Adipokinetic Hormone Receptor Sequence Alignment

We aligned the amino acid sequences of the AKHR from six lepidopteran genera (moths and butterflies). The AKHR sequences were obtained from BLAST searches. The multiple sequence alignment with hierarchical clustering was performed by using MultAlin version 5.4.1 (20).

## Results

### The Biological Effect of the N-terminus of Lacol-AKH in *H. eson*

AKH peptides are characterized by a pGlu in position 1. This is believed to be an effective block against exopeptidases in the hemolymph of the insect, resulting in a longer half-life of the peptide (21). Lacol-AKH analogs were designed to specifically explore how lipid mobilization is affected by changes to the N-terminal amino acid of the decapeptide. The results are shown in Table 2. When the N-terminal pGlu is replaced with a free Glu residue, the adipokinetic activity of the ligand was severely reduced from 100% to a mere 23% activity *in vivo*, whereas a free Gln residue in position 1 still had a strong biological effect of over 60% AKH activity relative to the maximal response. In contrast, lipids were not mobilized in *H. eson* when the N-terminal pGlu is replaced with a differently blocked Glu residue (viz. an acetylated Glu), or when a free Ala residue replaced pGlu in position 1.

### The Significance of N-terminally Shortened Lacol-AKH Analogs

Functional AKHs are either composed of eight, nine, or 10 amino acids; *H. eson* synthesizes AKHs with all of these chain lengths and all of these peptides were found to be biologically active in a conspecific bioassay (1). The biological effect of shorter chain lengths (6- and 7-mer) was previously explored with C-terminally truncated, amidated Lacol analogs (2). Here, the relative importance of the first three N-terminal residues are specifically examined in *H. eson* with Lacol-AKH analogs that have one absent residue (either pGlu^1^, Leu^2^, or Thr^3^), effectively producing an amidated nonapeptide.

None of the four nonapeptide Lacol-AKH analogs could achieve a high increase in circulating lipid concentrations and the AKH activity relative to Lacol-AKH injection in *H. eson* was well below 40% (Table 3). Without pGlu^1^, the peptide analog now beginning with a free Leu amino acid residue was 35% active, and this activity was further decreased to 12% when the Leu residue was blocked by N-acetylation. The return of pGlu^1^ coupled with the absence of Leu^2^ or Thr^3^ as analogs resulted in 36 and 25% activity, respectively (Table 3).

**Table 3:**
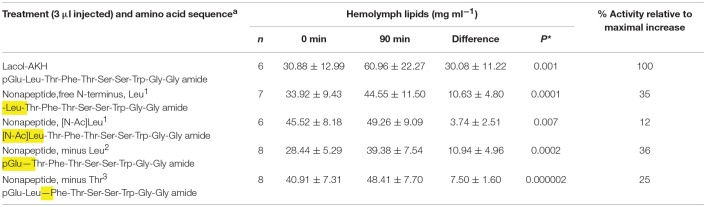
The biological effect of N-terminally shortened Lacol-AKH analogs in the common striped hawk moth, *Hippotion eson*.

^a^Amino acid substitutions or deletions in Lacol-AKH analogs are highlighted. The data are presented as Mean ± S.D. ^*^Paired t-test was used to calculate the significance between pre-and post-injection values.

### The Biological Effect of Single Amino Acid Replacements in Lacol-AKH

The relative importance of the middle region (amino acids 4–8) of Lacol-AKH was investigated via a series of analogs in which one amino acid was substituted with an alanine residue (Table 4). In this way we can make inferences about the relative importance of the side chains of the original C-terminal residues in activating the hawk moth AKH receptor. The substitution of an aromatic amino acid residue with Ala (i.e., Phe^4^ or Trp^8^) totally eliminated AKH activity (Table 4). Ala^6^ instead of Ser^6^ resulted in a marked reduction of biological activity (25%), whereas an Ala substitution in position 5 or 7 were more tolerated with 95 and 67% relative activity, respectively (Table 4).

**Table 4:**
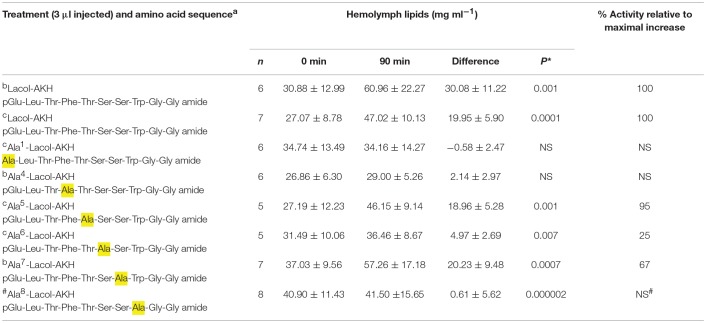
The biological effect of single amino acid substitutions in Lacol-AKH in the common striped hawk moth, *Hippotion eson*.

*^a^Amino acid substitutions in Lacol-AKH analogs are highlighted*.

Two data series (^b^ and ^c^) are presented as Mean ± S.D. The activity of analogs in a particular series was compared with the activity of Lacol-AKH of that series only. *Paired t-test was used to calculate the significance between pre-and post-injection values. NS, not significant.

*^#^Data from Marco and Gäde (2)*.

## Discussion

Previous studies showed that all five identified AKHs of the common striped hawk moth, *H. eson*, are active in conspecific biological assays to significantly increase lipid concentrations in the hemolymph (1). This indicated a degree of tolerance in the hereto unknown *H. eson* AKH receptor for accommodating (i) differences in peptide chain length of octapeptides (Hipes-AKH-I and -II), nonapeptides (Hipes-AKH-III and Manse-AKH), and a decapeptide (Manse-AKH-II; for peptide structures see Table 1), as well as (ii) for particular amino acid substitutions in positions 5 (the polar and neutral Ser or Thr) and 7 (Ser, Thr, or Gly) of the native AKHs. When a decapeptide AKH from a noctuid moth (Lacol-AKH) was 100% active in *H. eson* but Lacsp-AKH, the decapeptide AKH from a water bug was not (1), it presented an intriguing case into unraveling the reasons for the loss of biological activity. Thus, began a study with Lacol-AKH as lead peptide to examine the effect on adipokinetic activity of each of the three Lacsp-AKH amino acids that differed from the *Hippotion* AKHs, viz. Val^2^, Asn^3^, and Pro^6^ (2): Pro^6^ instead of the conserved *Hippotion* Ser^6^ made very little difference to adipokinetic activity, while Val^2^ or Asn^3^ in the place of the conserved Leu^2^ and Thr^3^ drastically curtailed biological activity in *H. eson*. Since Lacol-AKH and Lacsp-AKH both have Gly^10^ instead of *Hippotion*'s Gln^10^, and since Lacol-AKH is as active as the endogenous *H. eson* decapeptide AKH (Manse-AKH-II), this substitution was not investigated. A different substitution at position 10 in Manse-AKH-II, namely, a bulky and basic Lys residue (K^10^-Manse-AKH-II), still had 75% hyperlipemic activity, suggesting that major differences in charge, size and side chain (Gly, Gln, Lys) does not affect activity much, and that the 10th residue is not essential for interaction with the *H. eson* AKH receptor and its activation (2). These earlier studies, thus, also indicated that the N-terminal amino acid residues are quite important for ligand function *in vivo*; however, these alone do not constitute a functional core peptide, as demonstrated by the much reduced activity of amidated C-terminally truncated Lacol-AKH ligands (6- and 7-mer peptides) (2). The current study evolved from the afore-mentioned work with *H. eson* and Lacol-AKH analogs to study more closely the structure-activity effect of the N-terminus and N-terminal residues in a lepidopteran AKH, and to investigate the role of side-chains and charge of amino acid residues in the mid- and C-terminal part of a lepidopteran AKH in biological activity. Such information is valuable for the consideration of producing biostable peptide mimetics that could bind or block AKH receptors specifically in pest insects, such as lepidopterans.

The *in vivo* adipokinetic assay employed in the current study is very robust and we have used it with other lepidopterans before, such as *Mamestra brassicae* (22) and *Pieris brassicae* (14). Despite a variable starting level of lipids in newly-emerged *H. eson* adult specimens (24 h or less after eclosion), clear increases in circulating lipid levels can be measured within individual moths with a number of Lacol-AKH analogs tested in the present study; the statistically significant adipokinetic responses fall broadly within two categories, relative to a maximum response that is elicited with the same dose of the lead peptide, Lacol-AKH: 20–40%, and above 60%.

### The Importance of the N-terminus of Lacol-AKH on Biological Activity in *H. eson*

Among the characteristic features of AKH peptides are their blocked termini: a pyroglutamic acid (pGlu) in position 1, and an amidated C-terminus. Such a blocked ligand is less susceptible to exopeptidases in the hemolymph of the insect, and therefore results in a longer half-life of the peptide to achieve its hormonal effect. In the current study, Lacol-AKH analogs were designed to specifically explore how lipid mobilization is affected by changes to the N-terminal amino acid. When the N-terminal pGlu was replaced with a free Glu residue, the adipokinetic activity of the ligand dropped to 23% activity *in vivo*; on its own, this result could be interpreted as affirmation that the N-terminally unblocked peptide was enzymatically degraded. What speaks against this interpretation alone, is the result that a free Ala residue in position 1 was not able to mobilize lipids in *H. eson* to the same extent as the free Glu^1^ analog, thus suggesting that the analog with the slightly hydrophobic Ala^1^ residue has a quite different active conformation than the analog with a polar and negatively charged Glu^1^ residue and, hence, ligand-receptor interaction is even further impaired. Alternatively, the data may be interpreted that the Ala^1^ analog is degraded much faster by exopeptidases than the Glu^1^ analog, and/or that Glu^1^ could be converted slowly to pGlu *in vivo* to still achieve the necessary conformation for binding and activating the AKHR. Furthermore, an analog with a differently blocked N-terminus (an acetylated Glu^1^) failed to mobilize lipids in *H. eson*, thus could not activate the AKHR significantly despite the peptide being blocked at the N-terminus to delay degradation [current study and (2)]. This result suggests that the acetyl analog does not have the requisite conformation for accessing the receptor binding site, and concurs with an earlier study where Lee et al. (23) found that an unblocked Glu^1^-AKH analog was more active than a N-[Acetyl]Glu^1^-AKH analog in locusts *in vivo*. Results on other insects also reported lower biological activity when pGlu was replaced with other blocked residues (24, 25). Most surprisingly, however, was the observation that a free Gln^1^ Lacol-AKH in the current study attained nearly two-thirds maximal biological activity. This is a strong indicator that Gln was converted to pGlu in the hemolymph either enzymatically by glutaminyl cyclases or spontaneously (26) and thus could delay peptide degradation, and activate the AKHR through a suitable conformation. Since Glu^1^ and Gln^1^ AKH analogs were injected into *H. eson* at the same concentration of 10 pmol, and Gln^1^ produced a three times higher biological activity than Glu^1^, we may deduce that Gln is the preferred amino acid residue for conversion to pGlu. It has long been known that N-terminal Glu and Gln residues of many biologically active peptides and proteins can form pGlu by intramolecular cyclization, and several years of experimental evidence, reviewed by Abraham and Podell (27), seemed to indicate that either glutamic acid (Glu) or glutamine (Gln) is the direct precursor of pGlu, depending on the experimental system. Cyclization is catalyzed by an enzyme that is called glutaminyl cyclase (QC), although this enzyme also catalyses the conversion of glutamate to pGlu, hence it is an effective glutamyl cyclase (EC) too (28). That Gln seems to be the preferred substrate for QC in insects and crustacean, seems to also be apparent from the amino acid sequence encoded by the mRNA for the AKH precursor sequences known to date [see for example, (4, 29)].

In conclusion, our study has definitively shown that the N-terminal pGlu residue is of crucial importance for the correct presentation of the AKH ligand to its receptor to achieve and maintain full biological activity.

### Importance of N-terminal Amino Acids on Biological Activity Deduced From N-terminal Shortened Analogs

Although nonapeptides bind well to the *Hippotion* AKH receptor and achieve full or 75% of biological activity [Manse-AKH and Hipes-AKH-III, respectively; (1)], distortion of the N-terminus by free or blocked Leu as residue 1, or even peptides with pGlu as N-terminus but missing Leu^2^ or Thr^3^, failed to result in more than mediocre (>36%) activity in the current study. As argued before (24, 30) it appears to be of great importance to have an alternating amphiphilic orientation of polar (pGlu^1^, Thr^3^, Thr^5^) and hydrophobic (Leu^2^, Phe^4^) amino acids in the 5 positions at the N-terminus to form a beta-strand (31–33). One other reason for the poor adipokinetic response (20–40 %) when interfering with the first three amino acids at the N-terminus, may be the direct consequence of shifting the aromatic amino acid from position 4 to position 3 in all our nonapeptide analogs tested in the present study. Phe^4^ seems to be essential in all AKHs as this is one of the conserved AKH features (see also section Importance of side chains of individual amino acids on AKH biological activity).

### Importance of Side Chains of Individual Amino Acids on AKH Biological Activity

It had previously been shown with *H. eson*, that (i) a change from Leu^2^ to another aliphatic polar residue with a shorter side chain, such as Val, negatively influenced the activity of an AKH analog, whereas biological activity was almost restored to full activity when the stereoisomer Ile^2^ was introduced, and (ii) replacement of the neutral Thr^3^ residue with another neutral amino acid, Asn^3^ resulted in very little bioactivity (2), thus suggesting the importance of the correct side chains at positions 2 and 3 in the ligand for interaction with the AKH receptor. A similar vital importance of these residues can be deduced from earlier studies on *in vitro* receptor activation in insects as well as in a crustacean AKH/RPCH system (4, 30). The aromatic amino acid residues at position 4 (Phe; this study) and position 8 (Trp; (2)) of AKHs are also essential, as was previously found in all other structure-activity and receptor studies of insects [for example: (24, 25, 30, 34)] and a crustacean (4). It is also not surprising then that all known naturally-occurring AKH ligands have an aromatic amino acid at position 4 (Phe or Tyr) and 8 (Trp) [see (35) review]. The replacement of a polar Thr^5^ with a slightly hydrophobic Ala^5^ in the AKH analog was very well-tolerated in the current study, as was the case in a crustacean system (4). In other insect systems, however, changes at position 5 of AKHs resulted in strong loss of activity probably due to no H-bonding of the missing hydroxylated side chain (24, 30). Residue 5 is supposed to be the cornerstone of a beta-turn commencing residues 5 to 8. Previous structure-activity studies with AKHs also revealed that residue 6 (Pro) is essential for activity in insect systems, whereas residue 7 (Asp, Ala) were shown to be not essential (24, 30). The lead peptide in this study, Lacol-AKH, as all other lepidoptera-specific AKHs bar one (viz. Bommo-AKH; Table 1) do not possess a Pro^6^ residue; if it is introduced, no reduction in biological activity is measured in *H. eson* (2), suggesting that either a beta-turn is not necessary for the ligand conformation to bind to the receptor or that Ser^6^ is also able to form such a turn although it is generally known that Pro is a preferred residue for this formation [see (34)]. The simple Ala at position 6 cannot provide such conformation and activity is, hence, quite reduced in the current study. Residue 7, as in other systems (4, 24, 30) appears not to be participating in any H-bonding or other interaction with the AKH/RPCH receptor and, hence, an Ala^7^ analog has full activity in *H. eson* in the current study.

### Lepidopteran AKHs and Their Cognate Receptors

**Figure 1:**
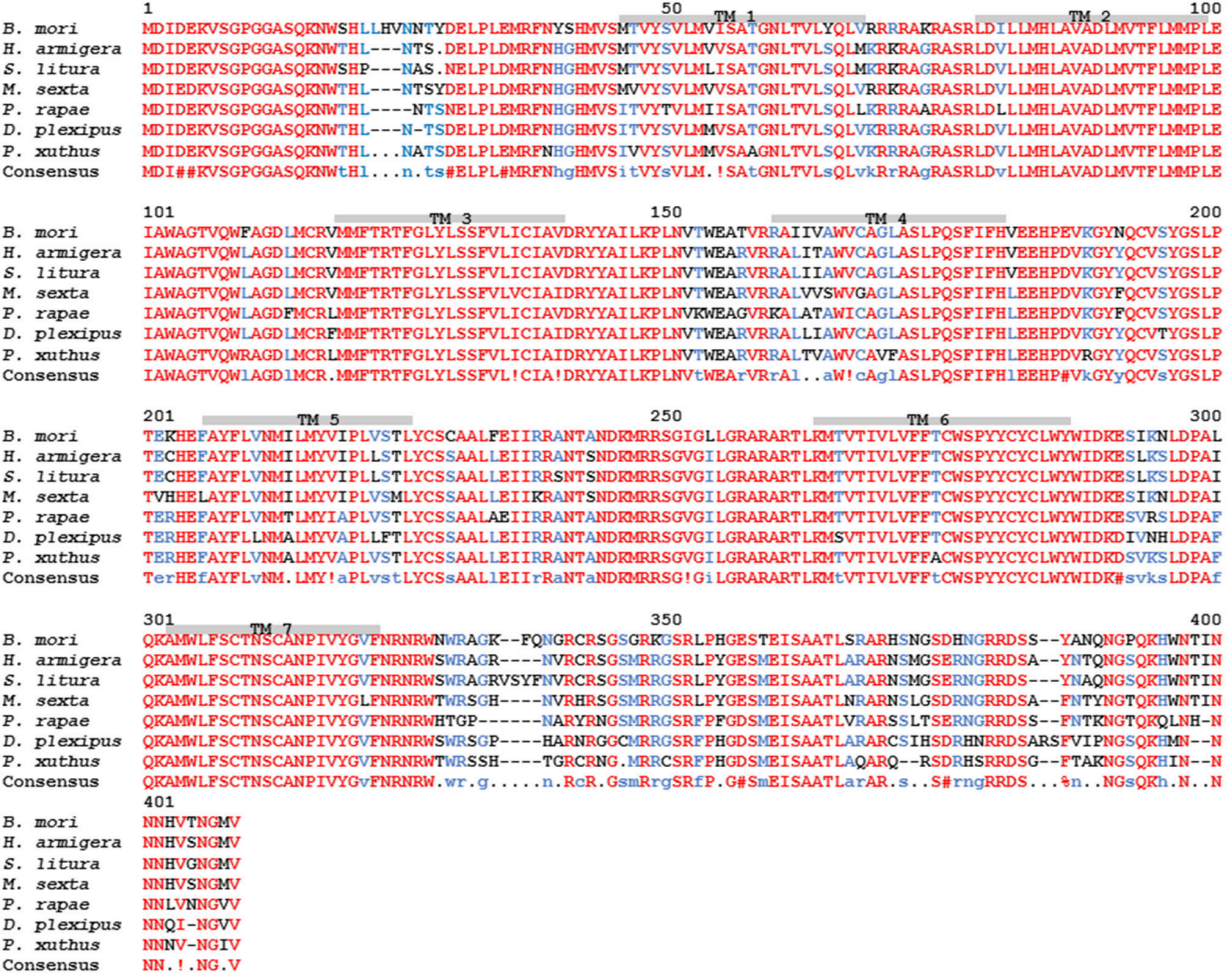
Amino acid sequence alignment of lepidopteran adipokinetic hormone receptor (AKHR) sequences. Moths: silk moth *Bombyx mori* = Bommo-AKHR (GenBank acc. no. **NP_001037049**), corn earworm moth *Helicoverpa armigera* (GenBank acc. no. **XP_021200838.1**), tobacco cutworm moth *Spodoptera litura* (GenBank acc. no. **XP_022815781.1**), and tobacco hornworm moth *Manduca sexta* = Manse-AKHR (GenBank acc. no. **ACE00761.1**). Butterflies: small white cabbage butterfly *Pieris rapae* (GenBank acc. no. **XP_022128572.1**), monarch butterfly *Danaus plexippus* (GenBank acc. no. **OWR46881.1**), and Asian swallowtail butterfly *Papillio xuthus* (GenBank acc. no. **XP_013163165.1**). The amino acid position is indicated above the residues. Identical residues between all the receptors are shown in red, and conservatively substituted residues in blue. Dashes indicate gaps that were introduced to maximize homologies. Putative transmembrane regions (TM1–TM7) are indicated by gray bars.

It is well-known from such model insect species as locusts and tobacco hornworm moth that AKHs are released into the hemolymph upon flight episodes (36, 37) and, from locusts, it is also known that the three endogenous AKHs are co-localized in the same secretory granules in the corpora cardiaca (CC) and, hence, released simultaneously and in the same ratio as found stored (38). By analogy, it is assumed that all five endogenous AKHs of *H. eson* are released together upon flight and in the same ratio as shown to occur in the CC [i.e., Hipes-AKH-I: Hipes-AKH-II: Manse-AKH: Hipes-AKH-III: Manse-AKH-II as 46: 30: 19: 8: 1; see Figure 2 of (1)]. As shown previously (1), all 5 AKHs are active and cause hyperlipaemia in the moth, thus the peptides must bind to a G-protein-coupled receptor (GPCR) to activate finally a lipase as reviewed previously (39). Do they all bind to the same receptor? Although the AKHR from a number of different insect species has been identified or predicted from genome sequencing projects [see, for example, (40, 41)], only one specific high-affinity AKHR has been found for each species, with the exception of some Diptera where various splice variants have been identified. The latter AKHR splice variants, however, do not have vastly different binding properties when examined experimentally (29, 42–44). Thus, it is postulated that there is only one AKHR for *H. eson* to which all five endogenous ligands bind. Although this receptor has not been cloned or data mined, there are other lepidopteran AKHRs characterized or genome-predicted. Figure 1 illustrates the alignment of seven butterfly/moth AKHRs. The consensus sequence clearly shows how similar these receptors are, and this is not only true for the transmembrane regions but for the extracellular loop regions as well. The region with the most differences in the lepidopteran AKH receptors spans about 10 amino acids after the seventh transmembrane domain (Figure 1).

To date, all AKHs occurring in Lepidoptera are unique to this order and have not been found in any other organism outside of this order. In *M. sexta* two AKHs have been identified, the nonapeptide Manse-AKH (10) and the decapeptide Manse-AKH-II (12). Both of these AKHs are also synthesized in the CC of *H. eson* (1), while the other 3 *H. eson* AKHs are slight modifications of Manse-AKH (see Table 1). As the AKHR of *M. sexta* is known (45), it should be possible in future to model the interaction of the 5 endogenous AKHs of *H. eson* with the *M. sexta* receptor, using similar methods as with ligand-receptor interaction studies on the AKH system of a dipteran insect, *Anopheles gambiae* (18), and the crustacean, *Daphnia pulex* (46). Once a model has been produced, the current structure-activity data of this study can be validated, and such a model can hopefully be used to narrow the search for agonists or antagonists, including peptidomimetics in order to utilize the AKH/AKHR system as order-specific “green insecticide.” Of paramount importance to this concept of “green” insecticides is the notion of specificity and discrimination of such compounds: thus, a pest insect should be harmed, but not a beneficial or mildly harmful insect. Thus, one of the next steps would be to do biological assays (or *in vitro* receptor-binding assays) to test the lepidopteran AKHs for cross-reactivity in non-pest insects. Such information would be critical for assessing whether there is any potential to further investigate the interaction of AKHs with the G protein-coupled receptor in lepidopterans to find a lead to an efficient “green” insecticide. The fact that the lepidopteran AKHs are unique to this order, may be an indication that the ligands are optimized for lepidopteran AKHRs, but this remains to be confirmed.

## Ethics Statement

This study was carried out in accordance with relevant institutional and national guidelines and regulations concerning the use of animal subjects in scientific studies. It is a University of Cape Town and national policy that the use of Insects are exempted from animal ethics applications. Nevertheless, the insects were handled in a proper veterinary manner.

## Author Contributions

HM and GG designed the research, collected animals (eggs and larvae), and wrote the manuscript. GG reared the moths from egg to adult stage. HM performed the experiments and analyzed the data. Both authors listed have approved the manuscript for publication.

### Conflict of Interest Statement

The authors declare that the research was conducted in the absence of any commercial or financial relationships that could be construed as a potential conflict of interest.
